# “Art is a guaranty of sanity,” Louise Bourgeois’ message for the COVID-19 pandemic!

**DOI:** 10.47626/2237-6089-2021-0261

**Published:** 2022-07-14

**Authors:** Philippe Courtet

**Affiliations:** 1 Department of Emergency Psychiatry and Acute Care Centre Hospitalier Universitaire Montpellier University of Montpellier Montpellier France Department of Emergency Psychiatry and Acute Care, Centre Hospitalier Universitaire Montpellier, IGF, University of Montpellier, CNRS, INSERM, F-34000 Montpellier, France.

After almost a year with several COVID-19 waves and related lockdowns, mental health is deteriorating everywhere in the world, as anticipated by many authors. Although the arrival of promising vaccines suggests some light at the end of the tunnel, the COVID-19 pandemic will continue for many months and its collective psychological impact may last even longer. Thus, mental health becomes a crucial issue and it is urgent to develop psychosocial support activities for everybody^1^ and yet the harsh health-related restrictions have particularly affected the world of culture. We may wonder about the paradox that arts, one of the activities essential for the well-being of the human species, are no longer available exactly when humanity is struggling against a devastating virus. As a matter of fact, from the beginning of the outbreak, people have been spontaneously sharing artistic activities on social networks, and museums have proposed many initiatives (e.g. #GettyMuseumChallenge). It is difficult not to see here an attempt by many individuals to adapt and cope with the major collective stress of the first lockdown.

We would like to underline the crucial role that arts can play in preserving mental health, raising the question of “arts on prescription.” Louise Bourgeois’ very rich artistic production during her long career can be used as an example to describe the many salutary aspects of the arts for the health and preservation of our society. The aim of this article is to discuss how Louise Bourgeois tried and succeeded to cope with major early stressors by producing art throughout her career. I would like to use Louise Bourgeois’ artistic production to highlight the inherent benefits of art practice for preserving mental health, by focusing on some of her major artworks that seem particularly in tune with different traumatic events related to the pandemic (i.e. feminist concerns, grief, abuse, confinement).

Louise Bourgeois (1911-2010), one of the most significant and influential contemporary artists, was the first woman to benefit from a retrospective at the MoMA in 1982. Through her art, she shared with the world the consequences of the childhood trauma related to her troubled family life that she obsessively relived. Her father was a tyrannical philanderer who had an affair with a governess, the discovery of which was the central trauma in Louise’ life. The death of her mother, who was the main caregiver figure for Louise, further complicated her youth. Of note, Louise Bourgeois started a 30-year-long psychoanalytic analysis soon after her father’s death because she had severe depression. Her whole art production became a way to process emotions, particularly pain and anger, and a lens through which she could examine her childhood experiences to create art that communicates powerful personal feelings through metaphors. Most artists include an autobiographical element in their work; however, in the case of Louise Bourgeois, it is important to recognize the high degree of sublimation she achieved in her work, reaching a timeless and universal status that refers more broadly to human consciousness and human perceptual experience. Moreover, her artistic production with the extreme “multi-layeredness” of her aesthetics did not focus on the art object as a result, but rather on the creative process itself, conceived as a journey with its own meaning and conceptual power. Traveling through Bourgeois’ art production allows the spectator to experience universal emotions, such as suffering and pain, anger and aggression, guilt and anxiety, depression and loneliness, with which she had to deal during her life, as a mean of connecting with the outside world. This is all in resonance with the current health crisis: “…within the family there was like a virus that was traumatic for us, for me, my brother and sister, we were suffering from a crack in the family unit…”.^2^

Importantly, Louise Bourgeois described her art as life-saving: “Art is a guaranty of sanity” she wrote on metal outside the sculpture-environment Precious Liquids, in 1992. She considered art as a form of mental mending, making art being empowering: “I need to make things. The physical interaction with the medium has a curative effect. I need the physical acting out. I need to have these objects exist in relation to my body.”^3^

This becomes clear when observing some of her iconic pieces. The imagery of housing, combined with female body shapes, dominated Bourgeois’ artistic production from the beginning of her artistic life with the Femme Maison (Woman House) series. In these works, from paintings to marble sculptures, she replaced the women’s heads with houses, isolating their bodies in the outside world without defenses and keeping their minds within the domestic walls. In this series, Bourgeois explored the female body/identity and home treated as one. Because of the visual and verbal play upon the term “housewife,” a feminist reading has been proposed for the Femme Maison series, which could depict the realities of a young mother confined at home with inescapable responsibilities. Such concerns are still topical as feminist struggles remain vivid. Moreover, the pandemic has reminded us about the reality of domestic violence and about the effects of home confinement for several months. Architecture could also symbolize the social world that attempts to define the individual, in contrast with the inner world of emotion. The tension between the human figure and architecture mirrors the dichotomy between mind and body. The polysemy of repeated images of isolation, disturbed communication, and loneliness of the Femme Maison series might embody intense feelings of helplessness, vulnerability, and fear. They are a reminder of the artist’s traumatic memories, but also bring to the fore the question of what kind of domestic space a person can produce and endure.

**Figure 1:**
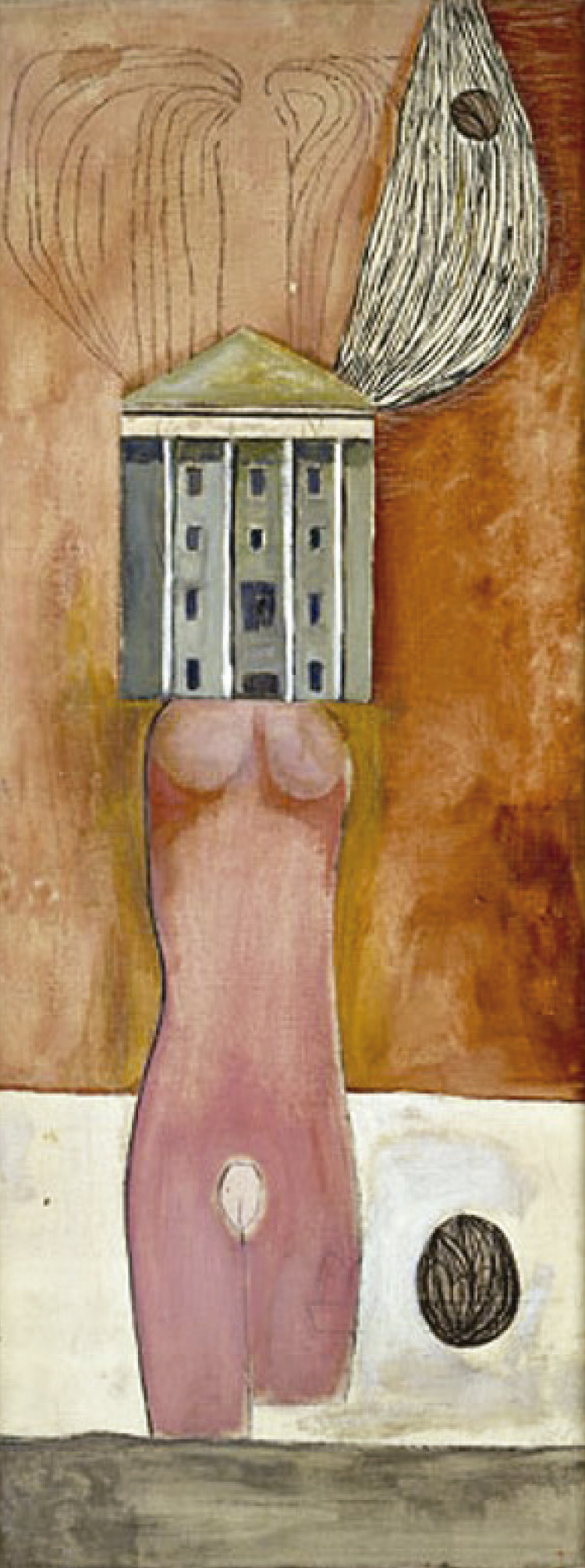
Femme Maison, Louise Bourgeois.

**Figure 2:**
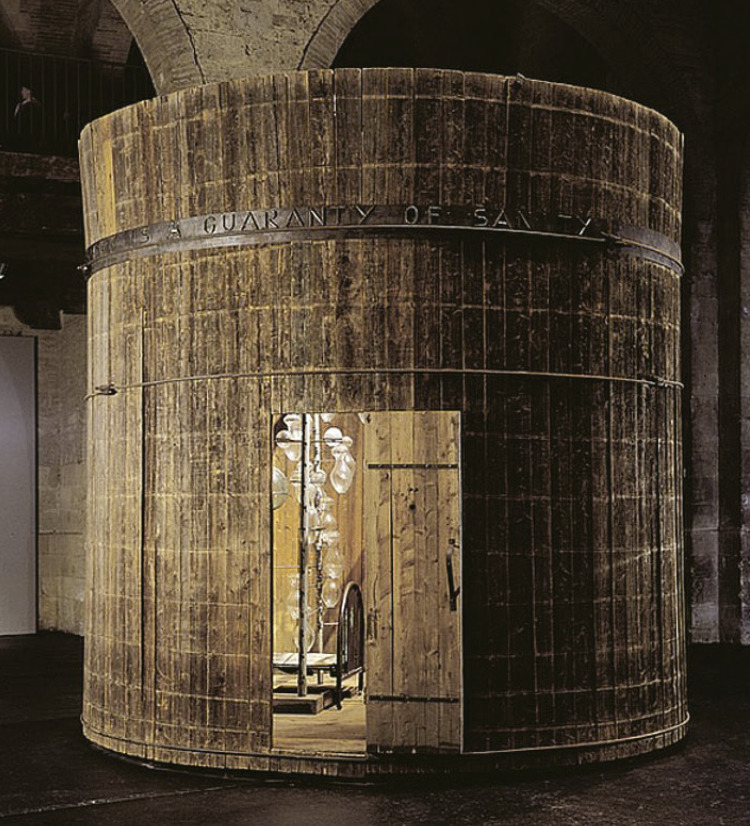
Cell, Louise Bourgeois.

After a long period working on “housewives,” Louise Bourgeois created “Cells,” installations exhibited at the Venice Biennale of Contemporary Art in 1993. Cells are enclosures constructed from a variety of scavenged materials, such as wire mesh and steel frames, old doors and windows, pieces of furniture, objects, sculptures, and sometimes fragmented body parts. The doors and windows that offer no way in or out create a feeling of confinement before the spectator’s eyes, and the insertion of mirrors inside the cell reinforces the obligation to perceive and come to terms with one’s body, one’s physical self, but also one’s psychological self. In these works, Bourgeois analyzed the body/emotion relationship, stating that the cells represent “*different types of pain; physical, emotional and psychological, mental and intellectual… Each Cell deals with a fear*.”^3^ Feeling of entrapments are aroused by the “Cells,” enclosed rooms, like a prison; however, the name “Cells” also evokes the body’s cells. The “Cells” series revisits the themes of confinement, anguish and fear, but also suggests new perspectives on reality. It is difficult not to grasp their powerful message in the current context, characterized by our monastic confinement at home, the aggression of our cells by the virus, and the hope that our immune cells will save us.

These artworks are particularly significant in the current context, a reminder of the wrenching presence of bodies in this period that has become entirely digital. The artist’s prescience also announced concepts that are becoming widely accepted in psychiatry, such as the importance of interoceptive pathways in the emotional experiences and the interactions of mind and body signals.^4^ Nor can we ignore the current questioning of the relationship between human beings and their architectural environment, with recent research showing a strong association between urbanicity and brain development and functioning.^5^ It is also striking that the combination of art and nature-based activities has synergistic effects that improve the psychosocial well-being of adult mental health service users.^6^ Importantly, the strong affective load, the organic metaphors, the constant presence of the body, and the interpersonal relationship games that characterize Bourgeois’ work inevitably convey empathy and compassion to the spectator. This can only challenge us in the current social, economic, and health crisis because altruism and social cohesion are essential for the resilience of our societies.

Bourgeois viewed her artistic creation as a therapeutic process to heal her losses and trauma. Similarly, art-based activities can help patients, regardless of their disease, to build a sense of self, to transform their illness experience into a positive experience, and to improve their well-being and quality of life. A growing body of evidence suggests that the arts have a role to play in health promotion. In the English Longitudinal Study of Ageing, Fancourt and Steptoe recently reported that the risk of death is reduced by 14-31% in people who engage with receptive arts activities, after controlling for demographic, socioeconomic, health-related, behavioral, and social factors.^7^ In a recent report, the World Health Organization^8^ identified the major role of arts in the prevention of mental problems, promotion of health and well-being, and disease management throughout life. Art engagement and creative art therapy can contribute to preventing and treating psychiatric disorders, including trauma-related illnesses. Specifically, art therapy is an experiential intervention that focuses on individual treatment goals through the therapeutic expression of personal experiences (memories, feelings, and emotions) using art materials, like painting, drawing, sculpting, and clay modelling.^9^ Interestingly, neuroscientific research suggests that arts trigger neural structures implicated in the regulation of body states (through biochemical, neuroendocrine, and neurocognitive processes), reversing the harmful inflammatory biological effects of social adversity and modulating affective state, with prosocial effects.^10^

The current COVID-19 crisis has created an unprecedented opportunity to recommend the development and study of artistic activities that can strengthen adaptation and resilience at the individual level and enhance social cohesion. This emphasizes the need for high-quality clinical trials to study the effectiveness of art therapy. Indeed, art therapy is considered an important supportive intervention in mental illnesses, but robust evidence on its effectiveness is currently lacking. In addition, as pressure on health resources grows, museums can be instrumental in offering holistic solutions to improve mental health. Investigations of such innovative public health practices should be encouraged to promote effective low-cost interventions for mental illness and social care.^11^ Arts on prescription interventions are part of the mainstream social prescribing resources in primary healthcare, and encouraging research on this topic will provide additional evidence on the value of such interventions for reducing anxiety and depression and increasing well-being.^12^

During the COVID-19 pandemic, we should not forget to engage in arts to improve and strengthen the health and well-being of our societies, as did Louise Bourgeois throughout her passionate and fruitful life.

## References

[1] Del Rio C, Collins LF, Malani P (2020). Long-term health consequences of COVID-19. JAMA.

[2] Bourgeois L (1998). Destruction of the father reconstruction of the father: writings and interviews 1923-1997.

[3] The art of Louise Bourgeois.

[4] Khalsa SS, Adolphs R, Cameron OG, Critchley HD, Davenport PW, Feinstein JS (2018). Interoception and mental health: a roadmap. Biol Psychiatry Cogn Neurosci Neuroimaging.

[5] Bratman GN, Anderson CB, Berman MG, Cochran B, de Vries S, Flanders J (2019). Nature and mental health: an ecosystem service perspective. Sci Adv.

[6] Thomson LJ, Morse N, Elsden E, Chatterjee HJ (2020). Art, nature and mental health: assessing the biopsychosocial effects of a ‘creative green prescription’ museum programme involving horticulture, artmaking and collections. Perspect Public Health.

[7] Fancourt D, Steptoe A (2019). The art of life and death: 14 year follow-up analyses of associations between arts engagement and mortality in the English longitudinal study of ageing. BMJ.

[8] Fancourt D, Finn S (2019). What is the evidence on the role of the arts in improving health and well-being? A scoping review.

[9] Haeyen S, van Hooren S, van der Veld W, Hutschemaekers G (2018). Efficacy of art therapy in individuals with personality disorders cluster B/C: a randomized controlled trial. J Pers Disord.

[10] Christensen JF, Gomila A (2018). Prog Brain Res.

[11] Tyrer P, Boardman J (2020). Refining social prescribing in the UK. Lancet Psychiatry.

[12] Sumner RC, Crone DM, Hughes S, James DVB (2021). Arts on prescription: observed changes in anxiety, depression, and well-being across referral cycles. Public Health.

